# Nuns as Nurses: Trained Nurses Superseded?

**Published:** 1919-09-20

**Authors:** 


### NURSING AFFAIRS IN IRELAND.

### Nuns as Nurses: Trained Nurses Superseded?

It is the fate of everything Irish to bear the brand of sectarianism. I knew a farmer who used to talk chaffingly about his "fine orange herd!" When a scavenger applies for a job under the municipality it is of greater importance to discover "with which foot he digs" (a euphemism for creed) than whether he is a good workman. Canvassing for an appointment is vigorously pursued on these lines, because an opposition candidate is always sure to appear.

### The Common Rule.

There is no exception to the common rule in the nurse's realm. If there is not a fair divide in non-sectarian institutions, there are jealousies and bickerings, but the orthodox plan is to make every institution sectarian, and to bar all comers who are, so far as each of them is concerned, nonconformist. It has been for many years the custom, where Roman Catholic patronage preponderates, to endeavour to introduce nuns—nursing sisters—to perform the duty. This has happened in many Poor-Law institutions. These women hold vows of poverty, and the moneys that they receive are, therefore, of no personal benefit to them. They are wage-earners for the Order to which they belong. Moreover, the immediate object is not nursing, nor indeed gain, but propaganda. I am not concerned with the polity of any church, but rather with nursing economics; and I think it is desirable to point out that the appointment of nuns as nurses in public institutions invariably prevents a trained Catholic nurse from earning her living. If the nuns were not in the field, Catholic patronage would operate in favour of a Catholic laywoman.

### Cases in Point.

Mrs. Mortished, Secretary to the Irish Nurses' Union, draws attention to the subject in a letter to the Irish Press. She points out that the nursing in the Dublin Corporation Tuberculosis Hospital was taken out of the hands of lay-trained nurses and given into the control of nuns. The same thing has happened in the Skin and Cancer Hospital, Dublin. The matron is retiring from the Crookaling Sanatorium, and a nun is to succeed her, when the whole staff will be replaced by nun sisters. Mrs. Mortished very truly observes that if the process is carried much further there will soon be no room left for the trained nurse in Ireland, unless she is a Protestant, "and that nursing, which, poorly paid though it be, is still one of the few professions open to Irish Catholic girls, will be closed to them." What happens in these cases, observes Mrs. Mortished, is not merely the appointment of a nun sister to fill an occasional vacancy in equal competition with lay nurses as to training, experience, etc., but the permanent introduction of a whole staff of nuns, the closing to the lay nurse avenues of promotion and threatening her with immediate or prospective unemployment.

### Another Aspect.

There is another aspect of this, which Mrs. Mortished does not mention. It happens that Catholic training-schools are particularly expensive. One of these in Dublin charges fifty guineas to probationers, and pays a salary during probation that is only nominal. It seems hardly playing the game to train her at such cost, and then to bestow her heritage on people who are not working for a career, and to whom nursing is a secondary consideration. I wish Mrs. Mortished success in her enterprise, but I am pessimistic as to the result. Catholic nurses will not enter into competition with the Church, for this is what it boils down to, and the tendency will be, as Mrs. Mortished portends, for well-educated Catholic girls to abandon the profession of nursing.

